# The Relationship Between Prenatal Heart to Heart Synchrony and Postnatal Mother-Infant Attachment and Behavior

**DOI:** 10.1192/j.eurpsy.2025.1147

**Published:** 2025-08-26

**Authors:** D. Ilki, L. Babayev, E. Sever, B. Ö. Konakçı, E. M. Karahan, B. Semiz, C. Ünal, M. Turğal, T. Mutluer

**Affiliations:** 1 School of Medicine, Koç University; 2 Child and Adolescent Psychiatry Department, Koç University Hospital; 3 Electrical and Electronics Engineering Department, Koç University; 4 Obstetrics and Gynecology Department, Koç University Hospital, Istanbul, Türkiye

## Abstract

**Introduction:**

Synchronization refers to the coordinated physiological, biological, and behavioral changes during interpersonal interactions.

**Objectives:**

The project aims to assess correlations between pre-term physiological synchrony and post-birth behavioral synchrony between mothers, fathers, and their anxiety, depression levels, and attachment styles. Since the development of early synchronization remains unclear, the project investigates its initiation between mother and fetus, with a focus on including fathers in early attachment and synchrony research. It is hypothesized that synchrony plays a key role in predicting a child’s attachment style.

**Methods:**

BIOPAC Student Lab MP36 measures ECG data from parents, while cardiotocography records the fetus’s heartbeat. Women in their 24-36th weeks of their first pregnancy without any chronic illnesses and their partners are being included in the study. Surveys for the participants cover sociodemographic scales, Beck Anxiety (BAI) and Depression Inventories (BDI) and The Relationship Scales Questionnaire. The recordings last fifteen minutes, with the first and last five minutes taking place in a non-stimulatory environment. During the middle five minutes, the fetus’s heartbeat is projected for the parents. ECG data are analyzed in Matlab for synchrony. At 3 months, parent-infant interactions will be videotaped and analyzed via Ruth Feldman’s *Coding Interactive Manual* for behavioral synchrony. Triads who show higher levels of physiological synchrony during pregnancy will be expected to show corresponding levels of behavioral synchrony at three months old.

**Results:**

The ECG and survey data of 16 participants have been collected. BAI results have shown the mean anxiety results of the mothers and the fathers to be 14.6 (mild anxiety), 4.9 (minimal anxiety), respectively, whereas BDI yielded mean depression results of 7.3, 6.3, both minimal depression for mothers and fathers. Out of 8 mothers, 4 showed secure and 4 showed dismissive attachment. 2 of the mothers with dismissive attachment showed moderate and severe levels of anxiety as expected whereas the other 2 mothers showed mild anxiety. The mothers with dismissive attachment showed higher anxiety levels and are expected to show lower physiological synchrony levels with their partners and babies. Among fathers, the most prevalent attachment style was secure, observed in 3 (37.5%), with the second being Dismissive attachment identified in 3 fathers (37.5%). One father exhibited a preoccupied/dismissive style, (12.5%)while one father showed a mixed secure/dismissive pattern(12.5%).

**Conclusions:**

The ECG data of the 16 participants are currently being evaluated for physiological synchrony between the triad and recruitments are still ongoing. After the infants are 3 months old, behavioral and physiological synchrony within the triads will be evaluated and analyzed for further relationships.

**Disclosure of Interest:**

None Declared

